# RNA-Seq Analysis and Gene Discovery of *Andrias davidianus* Using Illumina Short Read Sequencing

**DOI:** 10.1371/journal.pone.0123730

**Published:** 2015-04-13

**Authors:** Fenggang Li, Lixin Wang, Qingjing Lan, Hui Yang, Yang Li, Xiaolin Liu, Zhaoxia Yang

**Affiliations:** 1 College of Animal Science and Technology, Northwest A&F University, Shaanxi Key Laboratory of Molecular Biology for Agriculture, Yangling, Shaanxi, 712100, China; 2 Yellow River Fisheries Research Institute, Chinese Academy of Fishery Science, Xi’an, Shaanxi, 710086, China; University of Western Australia, AUSTRALIA

## Abstract

The Chinese giant salamander, *Andrias davidianus*, is an important species in the course of evolution; however, there is insufficient genomic data in public databases for understanding its immunologic mechanisms. High-throughput transcriptome sequencing is necessary to generate an enormous number of transcript sequences from *A*. *davidianus* for gene discovery. In this study, we generated more than 40 million reads from samples of spleen and skin tissue using the Illumina paired-end sequencing technology. *De novo* assembly yielded 87,297 transcripts with a mean length of 734 base pairs (bp). Based on the sequence similarities, searching with known proteins, 38,916 genes were identified. Gene enrichment analysis determined that 981 transcripts were assigned to the immune system. Tissue-specific expression analysis indicated that 443 of transcripts were specifically expressed in the spleen and skin. Among these transcripts, 147 transcripts were found to be involved in immune responses and inflammatory reactions, such as fucolectin, β-defensins and lymphotoxin beta. Eight tissue-specific genes were selected for validation using real time reverse transcription quantitative PCR (qRT-PCR). The results showed that these genes were significantly more expressed in spleen and skin than in other tissues, suggesting that these genes have vital roles in the immune response. This work provides a comprehensive genomic sequence resource for *A*. *davidianus* and lays the foundation for future research on the immunologic and disease resistance mechanisms of *A*. *davidianus* and other amphibians.

## Introduction

The Chinese giant salamander, *Andrias davidianus*, the largest amphibian on earth, may represent the transitional type of animal from aquatic to terrestrial life. The salamander is widely distributed in central and southern China [1]. However, due to overfishing and a long-term neglect of protection of natural resources, the wild populations of this animal have dramatically declined, making it an endangered species. Now, *A*. *davidianus* is estimated to be critically endangered by the International Union for Conservation of Nature and Natural Resources, and is a class II state major protected species in China [2]. Due to its weak migration ability, it depends on the aquatic environment. Throughout its evolution, it has developed a specific immune system enabling it to be adapted to this type of environment, making it an important model species for research in the fields of evolution and phylogeny.

Amphibians have developed a sophisticated immune system to adapt to the local environment compared with other aquatic animals. Both the skin and the spleen are immune organs in amphibians. Amphibian skin is rich with biologically active compounds, such as biogenic amines, complex alkaloids, and peptides, which are produced by holocrine-type serous glands in the integument. These active compounds are stored as granules in the lumen of the cells and are released upon stimulation. Therefore, amphibian skin, belonging to the non-specific immune defense system [3,4,5,6], is considered to be the first line of defense against external pathogenic microbes or predators. The spleen, the largest lymphoid organ of a vertebrate, can remove old erythrocytes from the circulatory system and lead to the efficient removal of blood-borne microorganisms and cellular debris. This function, in combination with a highly organized lymphoid compartment, makes the spleen the most important organ for inducing antibacterial and antifungal immune responses [7]. Studies on the immunologic mechanisms of *A*. *davidianus* or the function of genes related to the immune response have been limited due to the lack of genomic resources for this species. To date, there are only 407 expressed sequence tags (ESTs) available from the National Center for Biotechnology Information (NCBI) database originating from *A*. *davidianus*. Extensive genomic and transcriptome sequence data must be generated to permit for future research to discover new genes related to the immune response and inflammatory reactions of *A*. *davidianus*.

With the development of next-generation sequencing techniques, high-throughput sequencing [8,9,10] is becoming an efficient method for generating large amounts of genomic data and also eases identification of genes that are differentially expressed in distinct tissues or cells, developmental stages, physiological conditions, or upon activation of the immune system, especially for non-model organisms [11,12,13,14,15]. Recently, with the increasing read length of the Illumina sequencing technique and the development of new computational tools, ‘short-reads’ can be assembled for transcriptome analysis [14]. Compared with traditional characterization of ESTs by Sanger sequencing, next-generation sequencing of the transcriptome (also called RNA-Seq) is capable of generating high-throughput reads at a relatively low cost. Such approaches are being utilized for research in many areas such as *de novo* transcriptome assembly, gene-associated marker development and genome scale expression profiling [16,17]. Transcriptome analysis using Illumina sequencing technology has been reported in many non-model organisms, such as radix ovata (*Radix balthica*) [11], pacific oyster (*Crassostrea gigas*) [12], channel catfish (*Ictalurus punctatu*s) [18,19,20], patinopecten yessoensis (*Mizuhopecten yessoensis*) [21] and japonica rice (*Apostichopus japonicas*) [22].

In this study, we conducted Illumina paired-end sequencing of two different *A*. *davidianus* immune organs (skin and spleen) to generate a mass of transcripts and to discover genes associated with the immune response and inflammatory reactions. To the best of our knowledge, this is the first study to characterize the complete transcriptome of *A*. *davidianus* through the analysis of large-scale transcript sequences generated from Illumina paired-end sequencing. These sequences will serve as a valuable resource for novel gene discovery, the development of molecular markers, gene mapping and comparative genomic studies.

## Materials and Methods

### Ethics statement

The Chinese Wild Aquatic Animal Protection Law stipulates that the state shall encourage scientific research work for protect animals, and the used of wild animals in scientific research is not restricted.

In this study, all the samples were the second generation of the farmed *A*. *davidianus* from the artificial breeding farms which had obtained the permission of aquatic wild animals domestication and breeding management from the fishery administration of the People's Republic of China. The second generation of the farmed *A*. *davidianus* can be used in scientific research unrestricted.

This study has been also reviewed and approved by the ethics committee of Northwest A&F University. The Northwest A&F University approved the sacrifice of an endangered species for scientific research.

The People's Republic of China and Northwest A&F University are both the designated number of Institutional Animal Care and Use Committee (IACUC).

All Illumina reads and assembled transcripts files are available from the NCBI Sequence Read Archive database (accession number(s) SRP048762).

### Animal material and RNA Isolation

Nine healthy Chinese giant salamanders of three ages (one-year-old, two-year-old, three-year-old) were collected from a farm in Liuba County, Shaanxi Province, China. Animals were anesthetized and sacrificed by dissection before sample collection. All surgery was performed under MS-222 anesthesia, and every effort was made to minimize suffering. The salamanders underwent surgery under deep anesthesia and did not awaken after the procedure and the surgery was a terminal procedure. Skin and spleen tissues were dissected and flash-frozen in liquid nitrogen and stored at -80°C until use. Total RNA was extracted using the Trizol reagent according to the manufacturer’s instructions (Invitrogen, Carlsbad, CA, USA). The total RNA concentration was quantified using UV spectrophotometry, and the quality of total RNA was checked by electrophoresis in 1.5% agarose gel. The RNA integrity score and quantity were determined using an Agilent 2100 Bioanalyzer (Agilent, Beijing, China) prior to cDNA synthesis. Beads linked to oligo-dT were used for the enrichment and purification of poly-adenylated (poly-A) mRNA.

### cDNA Library Preparation for Sequencing

The purified mRNA (5 μg) was fragmented with divalent cations under increased temperature (60°C, 70°C, 80°C). These short fragments were utilized as template to synthesize first-strand cDNA using random hexamer primers and Superscript III (Invitrogen, Carlsbad, CA, USA). Second-strand cDNA was then synthesized in a solution containing buffer, dNTP, RNaseH and DNA polymerase I, and was subsequently purified using a QIAquick PCR extraction kit. EB buffer (elution buffer) was used to resolve these short fragments for end reparation and poly (A) addition. The sequence adaptors were linked to two ends of short cDNA sequences. Suitably sized cDNA fragments (20 bp) were selected for PCR amplification using agarose gel electrophoresis. Paired-end libraries, with approximate average insert lengths of 200 bp, were constructed using the Genomic Sample Prep kit (Illumina, San Diego, CA) according to manufacturer’s instructions. Prior to cluster generation, library concentration and size was assayed using the Agilent DNA1000 kit (Agilent, USA) on a 2100 Bioanalyzer (Agilent, Beijing, China). Libraries were sequenced as 101-mer×2 on an Illumina HiSeq 2000 sequencer equipped with a paired-end module at the BIOMARKER Biotechnology Co. (Beijing, China).

### Illumina Reads Processing and Assembly

A Perl script was written to remove low quality sequences (reads with a base quality less than 20) before the *de novo* transcriptome assembly. Then, the high quality reads were assembled using Trinity [23] with default settings, except for the K-mer value, to construct unique consensus sequences.

### Gene Annotations and Classifications

Functional annotations were carried out using sequence comparison with public databases. All unigenes were compared with the NCBI non-redundant nucleic acid database (NT), the NCBI non-redundant protein database (NR http://www.ncbi.nlm.nih.gov/), the Swiss-Prot database (http://www.expasy.ch/sprot), and the Clusters of Orthologous Groups database (http://www.ncbi.nlm.nih.gov/COG/) using BLAST with an E-value less than 1e^-5^. InterPro domains were annotated using InterProScan Release 16.0 and functional assignments were mapped onto Gene Ontology (GO) (http://www.geneontology.org/). Then, we used WEGO to perform GO functional classification of all unigenes in order to understand the distribution of gene functions at the macroscopic level (http://wego.genomics.org.cn/cgi-bin/wego/index.pl). Pathway assignments were carried out based on the KEGG database (http://www.genome.jp/kegg). Unigenes were compared with the KEGG database using BLASTX with an E-value less than 1e^-5^ and then a Perl script was developed to retrieve KO (KEGG Orthology) information from BLASTX results to establish that these transcripts were present in the pathway.

### Unigene Differential Expression Analysis

Using the number of reads mapped to the unigenes to calculate the expression levels of the unigenes is unreasonable statistically because the number of reads sampled from long unigenes is greater than that sampled from shorter unigenes. RPKM (reads per kilobase of transcript per million reads mapped) is the standardized method for calculating the expression level of a gene by normalizing the length of the unigenes. It is expressed as:

$$RPKM=\frac{10^{6}C}{NL/10^{3}}$$

where RPKM is the expression of a unigene X, C is the number of reads that is uniquely aligned to unigene X, N is the total number of reads that is uniquely aligned to all unigenes, and L is the base length in the coding domain sequence (CDS) of the unigene X [24]. The differential expression analysis of the spleen and skin tissues was carried out using IDEG6 software, which generates a generalized chi-square test where the P-value is corrected by multiple hypothesis testing. After correction, we considered a P-value less than 0.01 and a RPKM ratio of more than 2-fold between the two tissues as differentially expressed genes (DEGs).

### Expression analyses of DEGs by real time PCR (qRT-PCR)

We utilized real-time fluorescence monitored quantitative reverse transcription polymerase chain reaction (qRT-PCR) with a CFX96 Real-Time PCR Detection System (Bio-Rad) to analyze the tissue-specific expression and differential expression between the different ages (1, 2 and 3-years old) for a number of specifically expressed genes that were selected for validation. Beta-actin (β-actin) was included as an internal reference gene to normalize the variations of input total cDNA template among samples. Real-time PCR amplification reactions were carried out in a final volume of 25μl, which contained 12.5μl 2×SYBR Premix Ex TaqTM II (TaKaRa), 1μl diluted cDNA template, 10.5μl PCR-Grade water, and 0.5μl of each primer. Samples were heated to 95°C for 1 min as initial denaturation, followed by 40 cycles of denaturation at 95°C for 15s, and annealing/extension at 54–60°C for 1 min. Melting curves were also plotted (60–95°C) in order to make sure that a single PCR product was amplified for each pair of primers. (Table 1). qRT-PCR data was exported as CT values, which were analyzed using CFX Manager software and the expression results were presented as mean ± S.D. One-way ANOVA for the qPCR experiments for multiple comparisons between the mean of samples.

**Table 1:** Primers for real time PCR.

**Gene**	**Primer sequence**	**Tm (**°C**)**
***cystatin A***	GCGGTGATAACTGCGTAA	56°C
	TAGGGAAGCGGTTCATAC	58°C
***cystatin B***	ACAGACGGTTGCTGGATA	54°C
	TCGCCAGTTTCTTGCTAC	56°C
***β-defensin***	CCCTGTCTGTTTCGTTCAT	56°C
	CACTTTGTCCGATTCTTGCT	58°C
***galectin-3***	CACTTTGTCCGATTCTTGCT	58°C
	TTTCCGCTCCACTTCAATCC	60°C
***Lymphotoxin-beta***	ACTCATCTGAGAGGGTGGAA	60°C
	CCTGGCAGTAGACGTAGTAGA	53°C
***CD5L***	GATGCACAGGGAAGGAATCA	55°C
	CGATACCAACGTCCTCTTTGT	56°C
***Ig lambda chain V-I region BL2***	TGAGGCCAATGAAGGGTATTT	57°C
	TCCATGCTTTGGCAGTATCA	58°C
***SLC40A1***	GGGTAGACAAGAACGCAAGA	60°C
	GATGTAGCAAGCGGTAAGAA	58°C
***β-Actin***	GCCATCAATCGTCCACCG	58°C
	CCGCATCAAGCACCAGAA	56°C

## Results

### Illumina Sequencing and de novo Assembly

**Fig 1:**
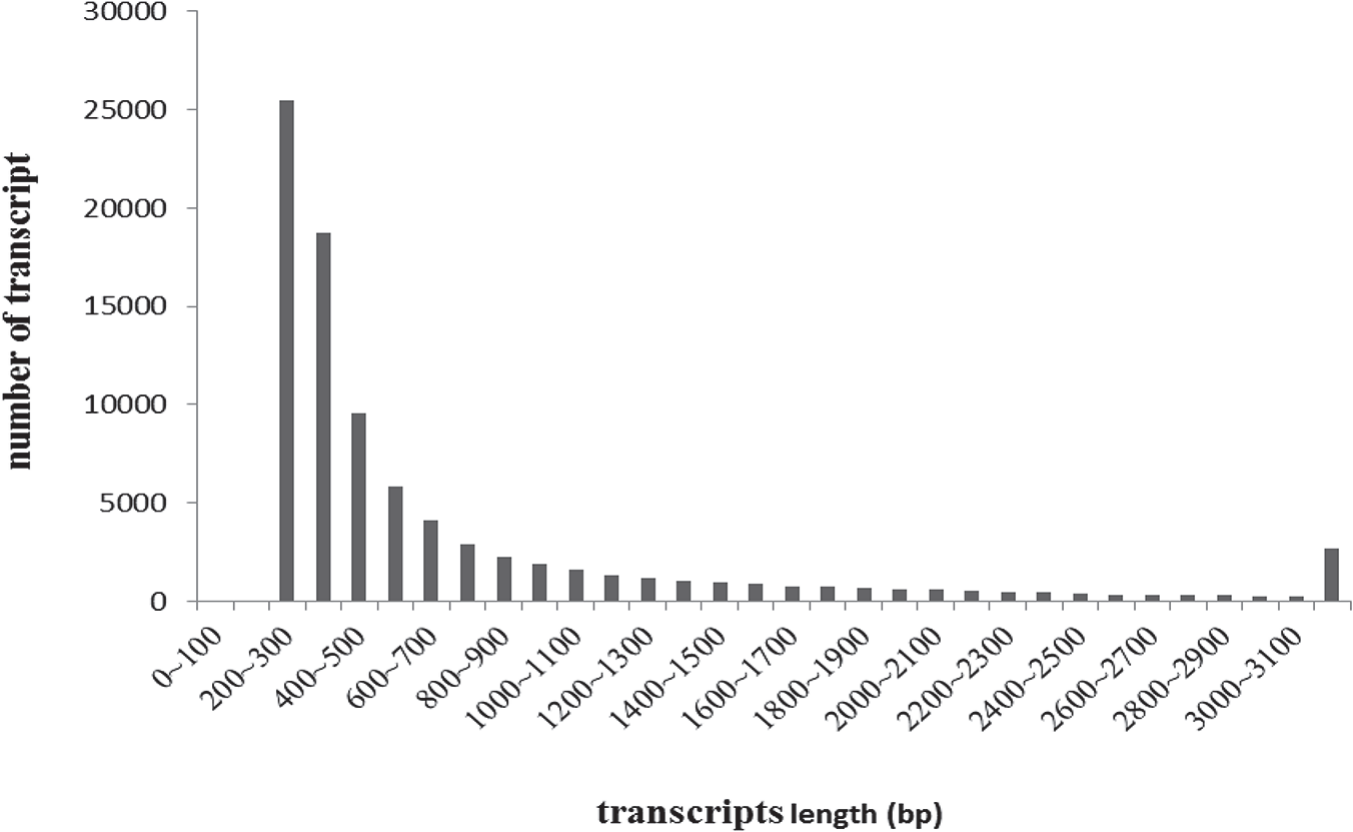
Length distribution of *Andrias davidianus* transcripts.

Illumina paired-end sequencing generated 20,587,272 and 20,698,732 raw reads from the spleen cDNA library (T1) and the skin cDNA library (T2), respectively. After stringent quality assessment and data filtering, reads with Q20 bases (those with a base quality greater than 20) were selected as high quality reads for further analysis. We used the Trinity program with an optimized K-mer length of 25 for *de novo* assembly. All short sequences were assembled into 1,495,015 and 2,161,318 contigs from the spleen and skin cDNA libraries, with N50 lengths of 117 bp and 91 bp, respectively. From these contigs, 85,173 and 73,891 scaffolds were constructed from the spleen and skin libraries, with mean lengths of 910 bp and 736 bp, respectively. Subsequently, 122,461 transcripts were obtained (64,807 transcripts with a mean length of 787 bp from spleen library and 57,654 transcripts with a mean length of 787 bp from skin library). All transcripts from both libraries were clustered according to sequence similarity, the longest sequence of each cluster was selected for downstream analysis. A total of 87,297 transcripts, with a mean length of 734 bp, was obtained by combining the two libraries (Table 2). As shown in Fig 1, the sequence length of these transcripts ranged from 200 bp to more than 3000 bp. The number of transcripts decreased with increasing length.

**Table 2:** Summary of transcripts from the spleen (T1), skin (T2) and the total transcripts in *A*. *davidianus*.

Total number (percentage)			
Length range	T1 transcripts	T2 transcripts	All transcripts
**200–300**	17,294 (26.69%)	16,670 (28.91%)	25,491 (29.20%)
**300–500**	20,467 (31.58%)	19,067 (33.07%)	28,272 (32.39%)
**500–1000**	13,106 (20.22%)	12,048 (20.90%)	16,935 (19.40%)
**1000–2000**	8,162 (12.59%)	6,673 (11.57%)	9,807 (11.23%)
**2000+**	5,778 (8.92%)	3,196 (5.54%)	6,792 (7.78%)
**Total number**	64,807	57,654	87,297
**Total length**	51,063,526	38,404,133	64,146,041
**N50 length**	1,350	965	1,216
**Mean length**	787.93	666.11	734.80

### Function Annotation and Discovery of Immune Genes

**Fig 2:**
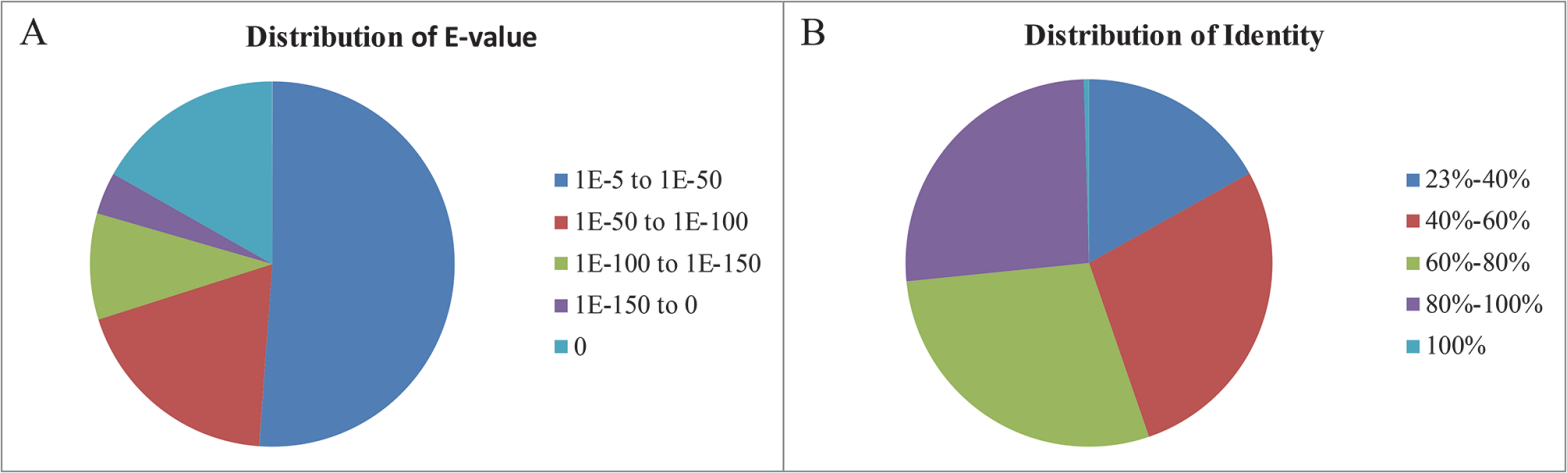
Characteristics of a similarity search of transcripts against Nr databases. (A) E-value distribution of BLAST hits for each transcripts with a cutoff E-value of 1.0E^-5^ in the Nr database. (B) Similarity distribution of the top BLAST hits for each transcripts in the Nr database.

For annotation and validation of the assembled transcripts, a sequence similarity search was conducted against the NCBI non-redundant protein (Nr) database and the Swiss-Prot protein database using the BLASTX algorithm with an E-value threshold of 1e^-5^. The results indicated that 34,972 of 87,297 (40.06%) transcripts showed significant similarity to known proteins in the Nr database. The E-value distribution of the top hits in the Nr database revealed that 48.84% of the mapped sequences showed significant homology (less than 1e^-50^) and nearly 26.6% of the sequences with greater than 80% similarity were observed (Fig 2). Similarly, we found that 28,608 (32.77%) had BLAST hits in the Swiss-Prot database. Notably, 12,836 (14.70%) transcripts with lengths over 1000 bp had BLAST matches, whereas only 8.56% of transcripts with lengths shorter than 300 bp had BLAST matches. Altogether, BLAST searches identified 38,916 unique protein accessions, indicating that a substantial fraction of *A*. *davidianus* genes were obtained in the present study (Table 3).

**Table 3:** Summary of annotations of assembled *A*. *davidianus* transcripts.

Annotated databases	All sequence	> = 300 bp	> = 1000 bp
**NR**	34,972	28,590	12,167
**NT**	21,937	19,336	10,111
**SwissProt**	28,608	24,309	11,312
**TrEMBL**	34,540	28,324	12,112
**GO**	25,904	21,963	10,588
**KEGG**	12,774	10,973	5,609
**COG**	7,709	6,828	4,009
**Total**	38,916	31,444	12,836

**Fig 3:**
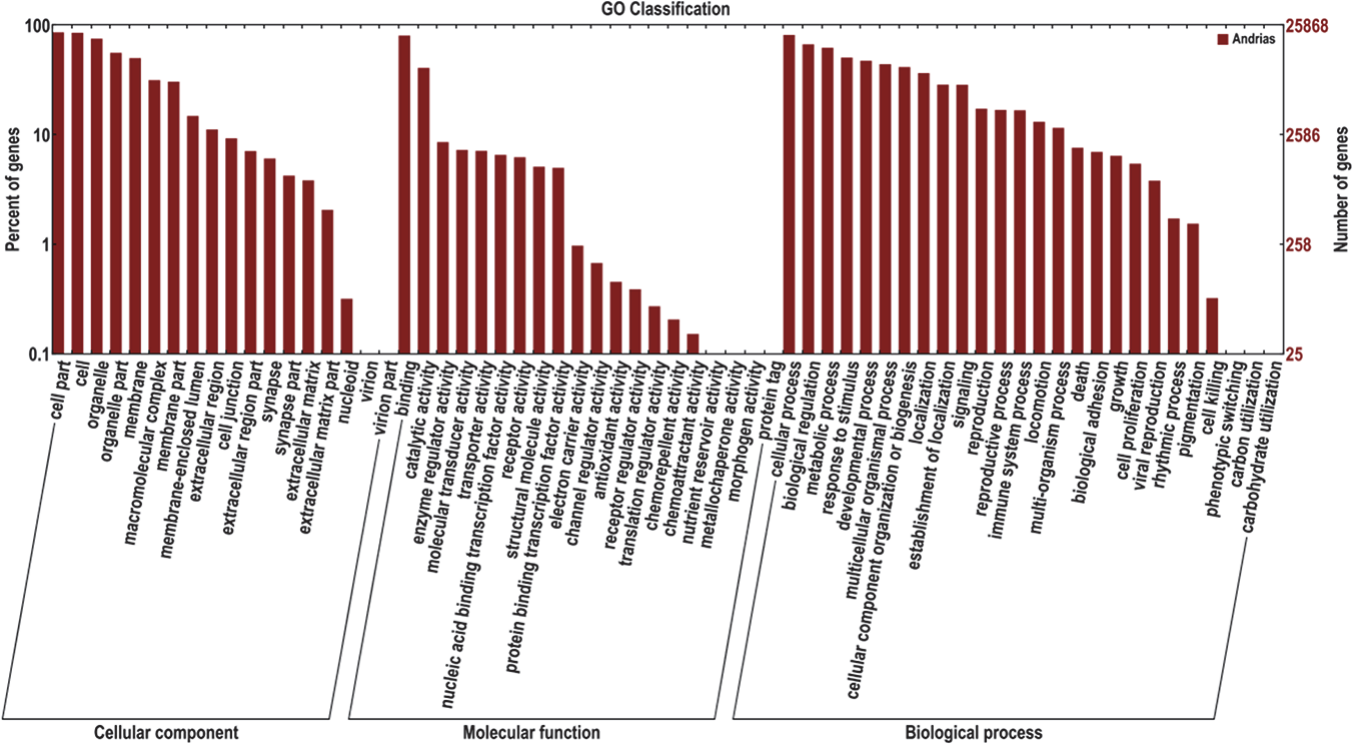
Gene Ontology Classification of assembled transcripts. The results are summarized in three main categories: (1) biological process, (2) cellular component and (3) molecular function. In total, 25,867 transcripts with BLAST matches to known proteins were assigned to gene ontology.

Gene Ontology (GO) is an international, standardized gene functional classification system which offers a constantly updated vocabulary and a strictly defined concept to comprehensively describe the properties of genes and their products in any organism. GO has three ontologies: (1) molecular function, (2) cellular component and (3) biological process. GO terms were subsequently assigned to *A*. *davidianus* unigenes based on their sequence matches to known proteins in the Nr database. A total of 25,867 transcripts (29.63%) were assigned at least one GO term, of which 9,713 (11.12%) were in the biological process category, 5,194 (6.77%) were in the cellular component category and 10,963 (12.47%) were in the molecular function category. Cellular processes (418 transcripts, 13.63%), cell part (18.18%) and binding (1,889 transcripts 18.18%) were the most abundant GO terms in the biological processes, cellular component and molecular function categories, respectively. Interestingly, a large number of genes (298 transcripts) involved in response to different stimuli were observed. A total of 153 transcripts were assigned to immune system processes. Under the category of molecular function, binding (1,889 transcripts, 17.23%) and catalytic (2,340 transcripts, 39.78%) functionalities represented the majority of this category. For the cellular component category, 2,850 transcripts were assigned to an intracellular location, whereas only a few genes were assigned to the extracellular compartment, macromolecular complex and virion modalities (Fig 3).

**Fig 4:**
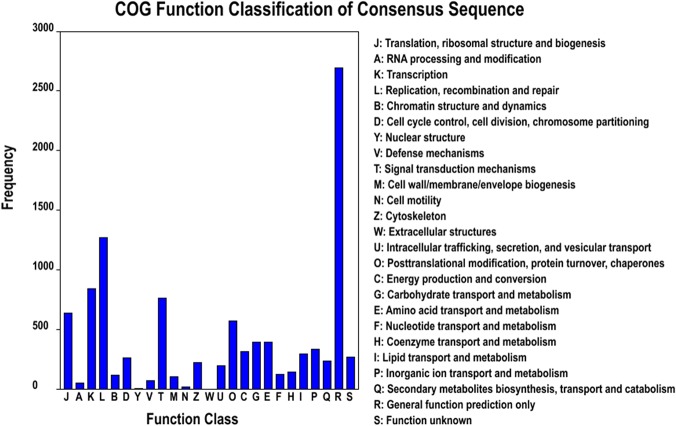
Histogram of clusters of orthologous groups (COG) classification. All transcripts were aligned to the COG database to predict and classify possible functions. Out of the 34,927 Nr hits, 7,709 sequences were assigned to 25 COG classifications.

The Clusters of Orthologous Groups of proteins (COGs) were delineated by comparing protein sequences encoded in complete genomes, representing major phylogenetic lineages. Each COG consists of individual proteins or groups of orthologs from at least 3 lineages and, thus, corresponds to an ancient conserved domain. All transcripts were aligned to the COG database to predict and classify possible functions. Out of the 34,927 Nr hits, 7,709 sequences were assigned to the COG classifications. Among the 25 COG categories, the cluster for general function prediction only (2,691 transcripts) represented the largest group, followed by replication, recombination and repair (1,270 transcripts), transcription (841 transcripts), signal transduction mechanisms (761 transcripts), translation, ribosomal structure and biogenesis (639 transcripts) and post-translational modification, protein turnover and chaperones (571 transcripts). Only a few transcripts were assigned to nuclear structure (15 transcripts) and extracellular structure (0 transcripts). In addition, 232 transcripts were assigned to secondary metabolites biosynthesis, transport and catabolism (Fig 4).

**Fig 5:**
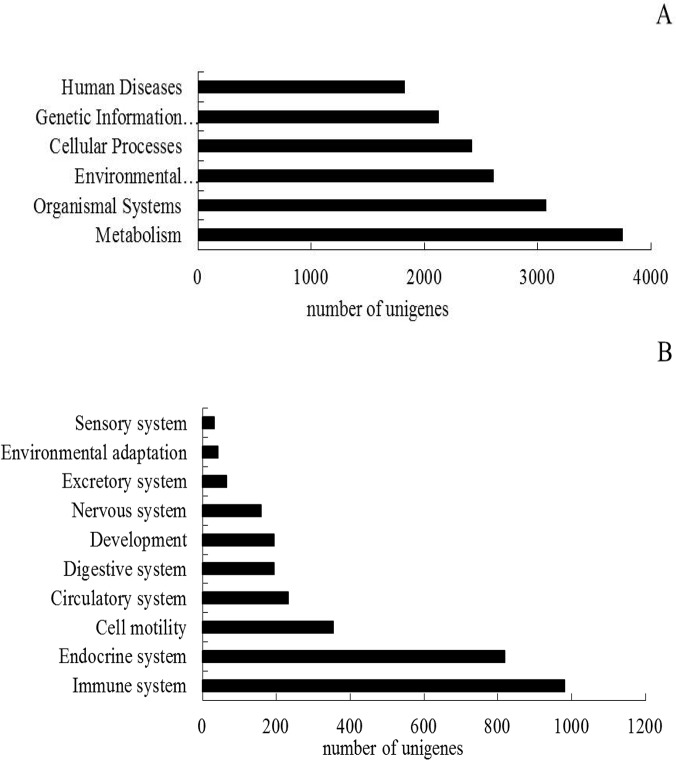
Pathways assignment based on KEGG. (A) Categories classified by KEGG; (B) Classification based on the organismal system.

The Kyoto Encyclopedia of Genes and Genomes (KEGG) Pathway database records the networks of molecular interactions in the cells and variants specific to particular organisms. Pathway-based analysis helps to further understand the biological functions and interactions of genes. First, based on a comparison against the KEGG database using BLASTX with an E-value cutoff of <10^–5^, 12,774 out of the 87,297 transcripts had significant matches in the database and were assigned to 243 KEGG pathways. The KEGG metabolic pathways were mainly carbohydrate metabolism, amino acid metabolism, lipid metabolism, energy metabolism and glycan biosynthesis and nucleotide metabolism. Of the 12,774 transcripts, 3,746 were assigned to metabolic pathways, composing the largest group of the seven categories classified by KEGG. Surprisingly, a large number of transcripts (981 transcripts) were assigned to the immune system pathway (Fig 5). The KEGG immune system pathway contained hematopoietic cell lineage, complement and coagulation cascades, toll-like receptor signaling pathway, nucleotide-binding oligomerization domain receptor signaling pathway and the retinoic acid inducible-gene I-like receptor signaling pathway.

### Tissue-specific Expression Analysis of Immune Genes

**Fig 6:**
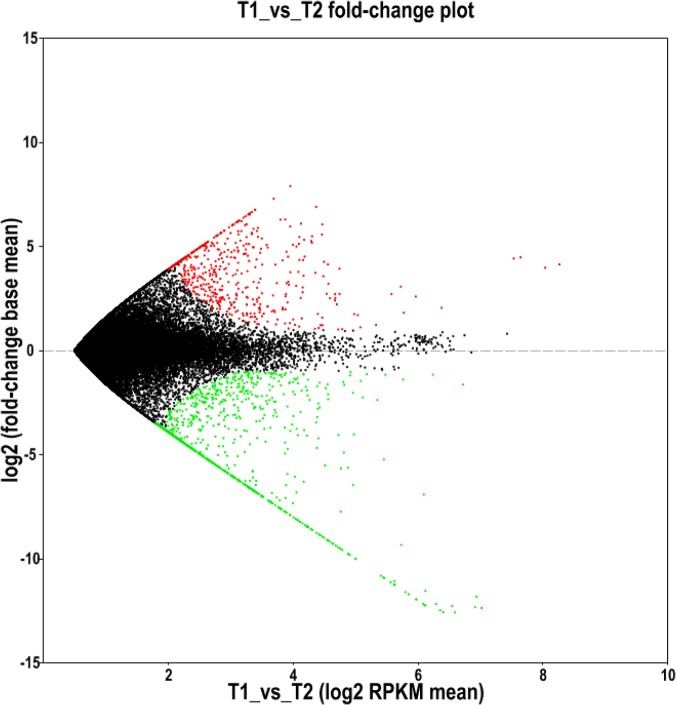
DEGs analysis of transcripts in the spleen and skin from *A*. *davidianus*. The red dots represent up-regulated genes; the green dots represent down-regulated genes; and the black dots represent non-significant differentially expressed genes.

**Fig 7:**
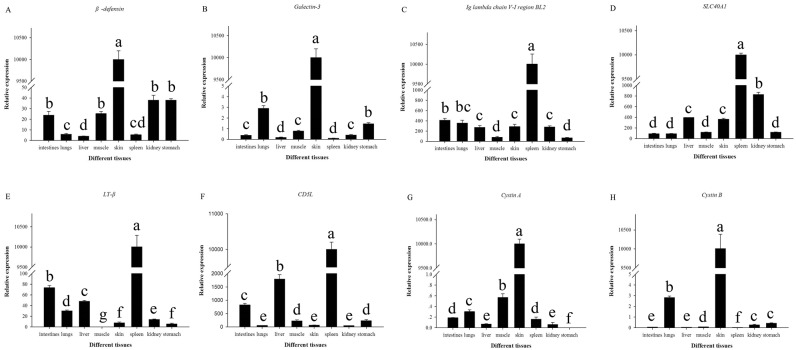
Tissue distribution of the selected genes of *A*. *davidianu*s in different tissues determined by quantitative real-time PCR. Different letters indicate statistically significant difference in relative expression of each gene among different tissues. Analyses were performed with SPSS Statistics 17.0.

**Fig 8:**
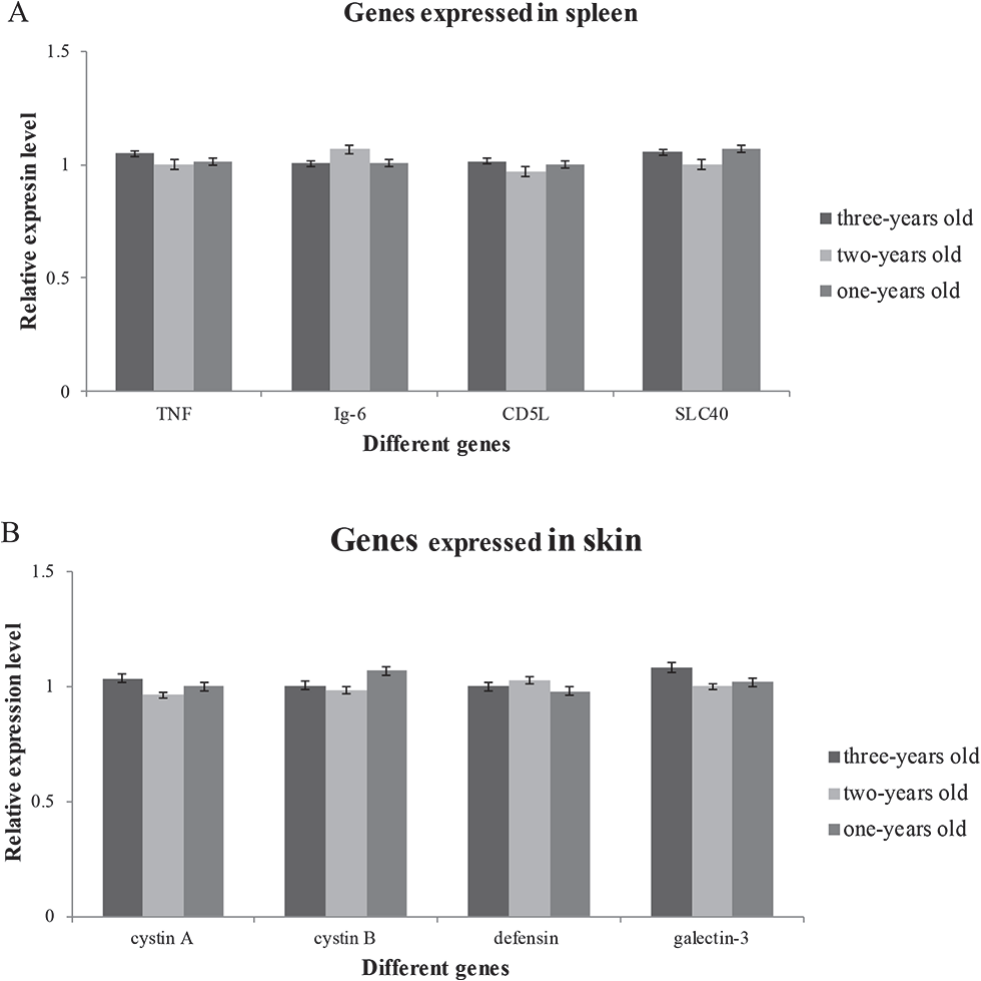
Expression patterns of the selected genes of *A*. *davidianus* at different ages determined by quantitative real-time PCR.

The differentially expressed genes identified in our transcriptome analysis of spleen and skin tissues in *A*. *davidianus* were investigated. Comparison of gene expression using the DEG method resulted in 518 transcripts that were expressed more in the spleen than in the skin, while 901 transcripts were down-regulated in the spleen compared with the skin (Fig 6). It was noteworthy that 110 transcripts were identified to be specifically expressed in the spleen, in contrast with 334 genes that were specifically expressed in the skin. We chose four skin-specifically-expressed genes (cytastin A, cytastin B, defensin, galectin-3 and four spleen-specifically-expressed genes (Lymphotoxin-beta, *SLC40A1*, Ig lambda chain V-I region BL2 and *CD5L*) to examine the tissue expression patterns in the skin, spleen, intestine, liver, muscle, kidney, lung and stomach using qRT-PCR. The results showed that all the four skin-specifically-expressed genes were all significantly more expressed in the skin compared with other tissues (p-value<0.05), while no expression was observed in the spleen. All of the four spleen-specifically-expressed genes were significantly more expressed in the spleen compared with other tissues (p-value<0.05), while only slight expression was detected in the skin (Fig 7). The expression patterns of the eight genes from three different ages (1-, 2- and 3-years old) were also analyzed. The result showed that no significant variation in expression was observed among the three age stages in both the skin and spleen for all the eight genes studied (Fig 8).

## Discussion

### Illumina paired end sequencing and assembly

Transcriptome sequencing is one of the most important tools for gene discovery; however, large-scale EST sequencing using the traditional Sanger method is time-consuming and expensive. During the past several years, RNA-Seq technology has become an advantageous approach for high-throughput gene discovery on a genome-wide scale in non-model organisms. In addition to its great improvements in efficiency and speed, RNA-Seq platforms can eliminate the bacterial cloning step that can bias the composition of the cDNA library [25]. However, accurate sequence reads and their reliable assembly is crucial for all downstream applications of RNA-Seq projects [26]. As the use of RNA-Seq continues to increase for non-model organisms, the need for *de novo* assembly algorithms concomitantly increases, especially for the assembly of ‘short-reads’ data for the Illumina platform. There are two alternative computational strategies for transcriptome reconstruction: the mapping-first approach, which is carried out based on an available reference genome, and the assembly-first (*de novo*) approach. The mapping-first approach promises, in principle, maximum sensitivity, but depends on correct read-to-reference alignment, a task that is complicated by splicing, sequencing errors and the lack or incompleteness of many reference or related genomes. Conversely, the assembly-first approach does not require any read-reference alignments, which is important for non-model species where the genomic sequence is not available [23]. Among the assembly-first methods, such as ABySS [27], SOAPdenovo [28], Trinity was proven to be a suitable method for the efficient and robust *de novo* reconstruction of transcriptomes [14,15,29,30,31,32]. Similarly to previous reports, our results also showed that short reads from Illumina paired-end sequencing could be well assembled and used for transcriptome analysis and gene identification in *A*. *davidianus*.

In this study, approximately 41,286,004 pair-reads (8.34 Gb) were generated using His-seq 2000 and were assembled into 87,297 transcripts with a mean length of 734 bp. The average length of the transcripts was longer than that of previous studies using other *de novo* assembly methods [11,25,33,34]. It is noteworthy that 16,599 transcripts (14.70%) had lengths longer than 1,000 bp, which is more than that of other organisms using Trinity for assembly [14,15]. These results showed Trinity is a powerful and efficient tool for *de novo* assembly for organisms without reference genomes. In addition, our study provided an abundance of expressed sequences, which will be valuable for further functional genomics research in *A*. *davidianus*.

### Functional annotation of unigenes

Due to the lack of an annotated reference genome, it is very difficult to predict the potential functions encoded by *A*. *davidianus* transcripts, thus we used the public protein databases combined with the BLASTX algorithm to annotate the assembled transcripts sequences. Our results indicated that 34,972 (40.06%) and 28,608 (32.77%) of the *A*. *davidianus* transcripts showed significant similarities to known proteins from the Nr and Swiss-Prot databases. A large number of sequences were assigned to a wide range of gene ontology categories and COG classifications. In addition, 12,774 transcripts were assigned into 243 pathways from the KEGG database including metabolism, organism systems, environmental information processing, cellular processes, genetic information processing and human diseases. Notably, 981 transcripts were consigned to 15 different subcategories of the immune system, such as leukocyte transendothelial migration, B cell receptor signaling pathway and the T cell receptor signaling pathway. These transcripts may be transcribed from genes responding to an antigen, which will be useful for a study of immunologic mechanisms. Although the unigenes without BLAST hits were likely derived from 5'- and 3'-untranslated regions, non-coding RNAs or short sequences not containing a known protein domain, they might represent potential *A*. *davidianus*-specific genes. Such a large number of sequences can provide a sufficient transcriptome sequence resource for discovering novel genes in *A*. *davidianus*. This study demonstrated that high-throughput Illumina paired-end sequencing is an efficient, inexpensive and reliable tool for transcriptome characterization and gene discovery in a non-model species.

### Tissue-Specific Immune Gene Discovery

As an organ that has direct contact with the environment, the skin of *A*. *davidianus* has gradually evolved complex defense strategies against invading microbes. The spleen is an important peripheral lymphoid organ, playing a crucial role in specific immune response processes. With regard to the tissue specific analysis of differentially expressed genes, numerous genes crucial for immune function and response to stimuli were identified. Using the DGE method to analyze differentially expressed genes between the two tissues studied resulted in the identification of 110 transcripts specifically expressed in the spleen and 334 unigenes specifically expressed in the skin.

Several genes of interest were identified in the skin, many of which are involved in biological activities related to defense against external pathogenic microbes or predators. Notably, a few genes are also associated with human disease. Lectins (hemagglutinins) are proteins which bind carbohydrates and agglutinate cells. Due to the diversity of their structure, they are classified into several protein families [35]. Their crucial biological role is defense against microorganisms, including phagocytosis, complement activation and the enhancement of natural killer cell activity. Some sequences were identified as members of the lectin superfamily in the present study. For instance, fucolectin, a lectin specific for α L-fucose [36,37] and also a Ca^2+^-independent non-glycosylated protein, was shown as a powerful defense agent against external microorganisms [37]. Galectin-3 contains a carbohydrate-recognition-binding domain (CRD) of approximately 130 amino acids that enable the specific binding of β-galactosides. Previous research implicated the expression of galectin-3 in a variety of processes associated with the induction of cell proliferation and the inhibition of cell apoptosis [38], tissue repair [39] and inflammation [40]. A sequence similar to β-defensins was also identified. The small (3–5 kDa) cationic defensins represent an important peptide family among the antimicrobial peptides. There are two superfamily members of defensins, α-defensins and β-defensins. Endogenous peptides, especially β-defensins, function against infection. They can destroy invading pathogens (including Gram-negative and Gram-positive bacteria, fungi and enveloped viruses) non-specifically [41]. Moreover, β-defensins also have cytotoxicity, which can inhibit the growth of tumor cells [42]. A previous study indicated that β-defensins 1 has been detected in salivary glands of an oral squamous cell carcinoma patient and some other defensins have also been found in the Langerhans cells which are adjacent to tumor cells. These defensins may be associated with the tumor and have a non-specific dose, time-dependent lethal effect [43]. Interestingly, a sequence similar to stonustoxin subunit alpha was also identified. *A*. *davidianus* skin is the first line of the innate defense system, where it can secrete massive amounts of bioactive molecules that defend against external pathogenic microbes or predators. Discovering these bioactive molecules in the present study is imperative in the development of new kinds of antibiotics and antimicrobials.

We also identified a number of spleen specifically-expressed transcripts which are similar to specific immune factors, such as lymphotoxin beta (TNF superfamily, member 3) and immunoglobulin light chain type III. Lymphotoxin beta (LTβ) is also known as tumor necrosis factor C, a member of the tumor necrosis factor family (TNF). LTβ anchors lymphotoxin-alpha (LTα) to the cell surface through the formation of a heterotrimer; the surface of an LTα-LTβ complex may have a specific role in immune regulation [44]. Studies have indicated that LT-β is present on a variety of lymphoid cell types including plasma cells and a subpopulation of CD4^+^ T cells in tissues affected by chronic inflammatory disease or infection. Therefore, LT-β may play a significant role in human disease and it may also represent a therapeutic target in a variety of common infective or inflammatory disorders [45]. The immunoglobulin light chain is the small polypeptide subunit of an antibody. There are two types of light chain in humans, kappa (κ) chain and lambda (λ) chain. For tetrapods, the immunoglobulin light chain genes can be classified into three distinct groups: kappa (κ), lambda (λ), and sigma (σ). The σ isotype was lost in the evolution of the amphibian lineage prior to the emergence of the true reptilian lineage [46]. The function of the immunoglobulin light chain is to provide antigen binding sites. To date, studies of light chains have focused predominantly on immunoglobulin light chains κ and λ and relevant results indicate that they are associated with human diseases. However, there have been few studies focusing on the σ isotype in amphibians. Therefore, it is imperative to investigate the biological function, molecular structure and the mechanism for regulating gene expression of the immunoglobulin light chain sigma (σ) isotype in order to thoroughly understand the mechanisms of this specific immune response.

### Tissue Specific Immune Gene expression analysis

To obtain a more thorough understanding of the potential biological roles of these immune factors, we analyzed the tissue specific expression patterns and the expression at three different ages. The expression of cystatin A, cystatin B, defensin, galectin-3, and fucolectin-5 were significantly up-regulated in the skin compared with other tissues, while only slightly expressed in the spleen. We conclude that these genes may play crucial roles in defense against external pathogenic microbes or predators. It was interesting that the four spleen specific genes were significantly expressed in the skin, but were only slightly expressed in other tissues. The Lymphotoxin-beta, CD5-*L*, Ig lambda chain V-I region BL2 and SLC40A1 are factors of adaptive immunity, therefore, they were significantly expressed in the spleen, but only slightly expressed in other tissues. However, these spleen-significantly expressed factors were also expressed in the skin. The reason for this may be that amphibian skin, not only has large number of bioactive molecules to defend against external pathogenic microbes or predators, but also has some immune globulins to participate in the adaptive immune system.

The expression levels of all eight immune genes examined did not show significant changes with ageing. This might imply that the immune system has already developed completely prior to *A*. *davidianus* becoming one year old. Due to severe environment stresses (the water temperature, water quality et.), *A*. *davidianus* may develop its immune system as early as possible in order to defend against external pathogenic microbes or predators.
